# [5-(4-Bromo­phenoxy­meth­yl)-1,3,4-thia­diazole-2-thiol­ato]triphenyl­tin(IV)

**DOI:** 10.1107/S1600536809012793

**Published:** 2009-04-18

**Authors:** Zhi-feng Wang, Gui-long Zhao, Lai-jin Tian

**Affiliations:** a School of Chemistry and Chemical Engineering, Qufu Normal University, Jining 273165, People’s Republic of China; b Tianjin Key Laboratory of Molecular Design and Drug Discovery, Tianjin Institute of Pharmaceutical Research, Tianjin 300193, People’s Republic of China

## Abstract

In title compound, [Sn(C_6_H_5_)_3_(C_9_H_6_BrN_2_OS_2_)], the Sn atom is five-coordinated and the 1,3,4-thia­diazole-2-thiol ligand acts as an *S*,*N*-bidentate chelating ligand. The five-coordinate Sn^IV^ atom forms four primary bonds, three to the phenyl groups and one to the S atom. Thus, the title complex has a distorted *cis*-trigonal bipyramidal geometry with the S atom and two C atoms occupying the equatorial plane, whereas the N atom and another C atom are in axial positions. In addition, there is a weak intramolecular Sn⋯N interaction. The crystal structure involves weak intra­molecular C—H⋯N and inter­molecular C—H⋯Br hydrogen bonding.

## Related literature

For the biological activity of 1,3,4-thia­diazole compounds, see: Oruc *et al.* (2004); Sawhney & Sharma (1993); Srivastava & Pandey (1993). For the biological activity of organotin(IV) compounds, see: Jimenez-Perez *et al.* (2000). For related crystal structures, see: Ma *et al.* (2006); Ng *et al.* (1990); Rodarte de Moura *et al.* (1999).
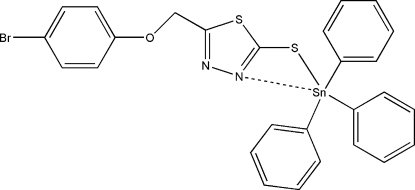


## Experimental

### 

#### Crystal data

[Sn(C_6_H_5_)_3_(C_9_H_6_BrN_2_OS_2_)]
*M* *_r_* = 652.18
Monoclinic, 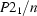

*a* = 15.524 (3) Å
*b* = 9.766 (2) Å
*c* = 18.019 (4) Å
β = 107.10 (3)°
*V* = 2611.2 (9) Å^3^
*Z* = 4
Mo *K*α radiation
μ = 2.69 mm^−1^
*T* = 293 K
0.22 × 0.20 × 0.16 mm

#### Data collection

Rigaku Saturn CCD area-detector diffractometer
Absorption correction: multi-scan (*CrystalClear*; Rigaku, 2005) *T*_min_ = 0.589, *T*_max_ = 0.673
17098 measured reflections
4610 independent reflections
3736 reflections with *I* > 2σ(*I*)
*R*_int_ = 0.034

#### Refinement

*R*[*F* ^2^ > 2σ(*F* ^2^)] = 0.034
*wR*(*F* ^2^) = 0.077
*S* = 1.04
4610 reflections
308 parameters
H-atom parameters constrained
Δρ_max_ = 0.54 e Å^−3^
Δρ_min_ = −0.38 e Å^−3^

### 

Data collection: *CrystalClear* (Rigaku, 2005); cell refinement: *CrystalClear* (Rigaku, 2005); data reduction: *CrystalClear* (Rigaku, 2005); program(s) used to solve structure: *SHELXTL* (Sheldrick, 2008); program(s) used to refine structure: *SHELXTL* (Sheldrick, 2008); molecular graphics: *SHELXTL* (Sheldrick, 2008); software used to prepare material for publication: *SHELXTL* (Sheldrick, 2008).

## Supplementary Material

Crystal structure: contains datablocks I, global. DOI: 10.1107/S1600536809012793/hg2497sup1.cif

Structure factors: contains datablocks I. DOI: 10.1107/S1600536809012793/hg2497Isup2.hkl

Additional supplementary materials:  crystallographic information; 3D view; checkCIF report

## Figures and Tables

**Table 1:** Selected bond lengths (Å)

Sn1—C13	2.130 (3)
Sn1—C7	2.146 (3)
Sn1—C1	2.149 (3)
Sn1—S1	2.4721 (10)
Sn1⋯N1	2.919 (3)

**Table 2:** Hydrogen-bond geometry (Å, °)

*D*—H⋯*A*	*D*—H	H⋯*A*	*D*⋯*A*	*D*—H⋯*A*
C9—H9⋯Br1^i^	0.93	2.87	3.627 (4)	139
C8—H8⋯N1	0.93	2.54	3.274 (5)	136

Symmetry code: (i) 
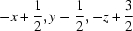
.

**Fig. 1.**
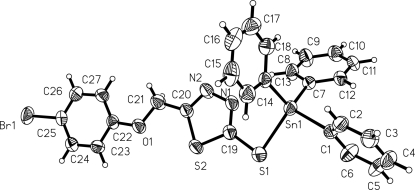
View of the title compound, with displacement ellipsoids drawn at the 40% probability level.

## supplementary crystallographic information

### Comment

1, 3, 4-thiadiazole compounds, an important class of intermediates in the medical and chemical syntheses, have attracted pharmacologist's interest in recent years due to their biological activity, such as antibacterial, antiviral, growth regulation and antitumoural activity (Oruc *et al.*, 2004; Sawhney & Sharma, 1993; Srivastava & Pandey, 1993). Meanwhile, organotins (IV) have been well known for their biological activities (Jimenez-Perez *et al.*, 2000). In order to find a new compound with broad spectrum of bioactivity we have designed and synthesized the title compound.

The title compound, bond lengths and angles are normal and in a good agreement with those reported previously (Ma *et al.*, 2006; Ng *et al.*, 1990). The five coordinated tin atoms forms four primary bonds: three to the phenyl groups and one to the sulfur atom. In addition, there is a weak intramolecular Sn···N interaction, the Sn1···N1 bond length (2.92 (2) Å) is longer than the sum of covalent radii (2.15 Å), but is shorter than that reported in Ph_3_Sn(MBZ) (3.07 Å) (Rodarte de Moura *et al.*, 1999). So the ligand 1,3,4-thiadiazole-2-thiol acts as a bidentate S, N chelating ligand. The crystal structure involves weak intramolecular C—H···N and intermolecular C—H···Br hydrogen bonding.

### Experimental

5-[(4-Bromophenoxy)methyl]-2-mercapto-1,3,4-thiadiazole (0.31 g, 1 mmol) and Ph_3_SnCl (0.385 g, 1 mmol) were dissolved in 30 ml of toluene, and the resultant mixture was heated to reflux for 6 h. The solvent was removed on a rotary evaporator, and the residue was heated in 50 ml of boiling dichloromethane/absolute ethanol (1/1 by volume). The solution was cooled to room temperature and then filtered, and the filtrate was evaporated slowly at room temperature, from which the crystals suitable for the X-ray diffraction were thus obtained.

### Refinement

All H atoms were found on difference maps, with C—H = 0.93 or 0.97 and included in the final cycles of refinement using a riding model, with *U*_iso_(H) = 1.2*U*_eq_(C).

### Crystal data

**Table:**

[Sn(C_6_H_5_)_3_(C_9_H_6_BrN_2_OS_2_)]	*F*(000) = 1288
*M**_r_* = 652.18	*D*_x_ = 1.659 Mg m^−^^3^
Monoclinic, *P*2_1_/*n*	Mo *K*α radiation, λ = 0.71073 Å
Hall symbol: -P 2yn	Cell parameters from 6604 reflections
*a* = 15.524 (3) Å	θ = 2.5–27.1°
*b* = 9.766 (2) Å	µ = 2.69 mm^−^^1^
*c* = 18.019 (4) Å	*T* = 293 K
β = 107.10 (3)°	Platelet, colorless
*V* = 2611.2 (9) Å^3^	0.22 × 0.20 × 0.16 mm
*Z* = 4	

### Data collection

**Table:**

Rigaku Saturn CCD area-detector diffractometer	4610 independent reflections
Radiation source: rotating anode	3736 reflections with *I* > 2σ(*I*)
confocal	*R*_int_ = 0.034
Detector resolution: 7.31 pixels mm^-1^	θ_max_ = 25.0°, θ_min_ = 2.5°
ω and φ scans	*h* = −18→18
Absorption correction: multi-scan (*CrystalClear*; Rigaku, 2005)	*k* = −11→11
*T*_min_ = 0.589, *T*_max_ = 0.673	*l* = −21→20
17098 measured reflections	

### Refinement

**Table:**

Refinement on *F*^2^	Secondary atom site location: difference Fourier map
Least-squares matrix: full	Hydrogen site location: inferred from neighbouring sites
*R*[*F*^2^ > 2σ(*F*^2^)] = 0.034	H-atom parameters constrained
*wR*(*F*^2^) = 0.077	*w* = 1/[σ^2^(*F*_o_^2^) + (0.0416*P*)^2^] where *P* = (*F*_o_^2^ + 2*F*_c_^2^)/3
*S* = 1.04	(Δ/σ)_max_ = 0.003
4610 reflections	Δρ_max_ = 0.54 e Å^−^^3^
308 parameters	Δρ_min_ = −0.38 e Å^−^^3^
0 restraints	Extinction correction: *SHELXTL* (Sheldrick, 2008), Fc^*^=kFc[1+0.001xFc^2^λ^3^/sin(2θ)]^-1/4^
Primary atom site location: structure-invariant direct methods	Extinction coefficient: 0.0068 (4)

### Special details

**Table:**

Geometry. All e.s.d.'s (except the e.s.d. in the dihedral angle between two l.s. planes) are estimated using the full covariance matrix. The cell e.s.d.'s are taken into account individually in the estimation of e.s.d.'s in distances, angles and torsion angles; correlations between e.s.d.'s in cell parameters are only used when they are defined by crystal symmetry. An approximate (isotropic) treatment of cell e.s.d.'s is used for estimating e.s.d.'s involving l.s. planes.
Refinement. Refinement of *F*^2^ against ALL reflections. The weighted *R*-factor *wR* and goodness of fit *S* are based on *F*^2^, conventional *R*-factors *R* are based on *F*, with *F* set to zero for negative *F*^2^. The threshold expression of *F*^2^ > σ(*F*^2^) is used only for calculating *R*-factors(gt) *etc*. and is not relevant to the choice of reflections for refinement. *R*-factors based on *F*^2^ are statistically about twice as large as those based on *F*, and *R*- factors based on ALL data will be even larger.

### Fractional atomic coordinates and isotropic or equivalent isotropic displacement parameters (Å^2^)

**Table:**

	*x*	*y*	*z*	*U*_iso_*/*U*_eq_	
Sn1	0.812768 (15)	0.08685 (2)	1.009453 (13)	0.04854 (12)	
Br1	−0.04987 (3)	0.06136 (5)	0.59342 (3)	0.08367 (17)	
S1	0.68434 (6)	0.19432 (11)	1.04208 (6)	0.0688 (3)	
S2	0.49137 (6)	0.15741 (10)	0.93629 (6)	0.0633 (3)	
O1	0.32794 (15)	0.1042 (2)	0.80766 (14)	0.0618 (6)	
N1	0.62663 (18)	0.0247 (3)	0.92206 (17)	0.0562 (7)	
N2	0.55438 (19)	−0.0210 (3)	0.86289 (18)	0.0596 (8)	
C1	0.9210 (2)	0.1827 (3)	1.09675 (19)	0.0473 (8)	
C2	0.9848 (2)	0.2580 (3)	1.0757 (2)	0.0584 (9)	
H2	0.9786	0.2713	1.0232	0.070*	
C3	1.0581 (2)	0.3144 (4)	1.1306 (2)	0.0705 (11)	
H3	1.1003	0.3656	1.1150	0.085*	
C4	1.0682 (3)	0.2949 (4)	1.2074 (2)	0.0729 (11)	
H4	1.1177	0.3320	1.2444	0.088*	
C5	1.0055 (3)	0.2207 (4)	1.2304 (2)	0.0775 (12)	
H5	1.0124	0.2072	1.2829	0.093*	
C6	0.9316 (3)	0.1658 (4)	1.1749 (2)	0.0674 (10)	
H6	0.8887	0.1170	1.1906	0.081*	
C7	0.8293 (2)	−0.1298 (3)	1.02802 (18)	0.0469 (8)	
C8	0.7640 (3)	−0.2272 (3)	0.9964 (2)	0.0621 (9)	
H8	0.7080	−0.2005	0.9642	0.075*	
C9	0.7824 (3)	−0.3646 (4)	1.0128 (2)	0.0737 (11)	
H9	0.7381	−0.4293	0.9914	0.088*	
C10	0.8638 (3)	−0.4068 (4)	1.0595 (2)	0.0667 (10)	
H10	0.8746	−0.4993	1.0705	0.080*	
C11	0.9300 (2)	−0.3114 (4)	1.0904 (2)	0.0592 (9)	
H11	0.9863	−0.3394	1.1214	0.071*	
C12	0.9121 (2)	−0.1734 (4)	1.0750 (2)	0.0548 (9)	
H12	0.9565	−0.1091	1.0968	0.066*	
C13	0.8166 (2)	0.1580 (3)	0.89873 (19)	0.0475 (8)	
C14	0.7985 (3)	0.2930 (4)	0.8760 (2)	0.0686 (10)	
H14	0.7800	0.3537	0.9080	0.082*	
C15	0.8078 (3)	0.3388 (4)	0.8055 (3)	0.0816 (13)	
H15	0.7954	0.4296	0.7908	0.098*	
C16	0.8350 (3)	0.2514 (5)	0.7582 (2)	0.0813 (13)	
H16	0.8418	0.2824	0.7114	0.098*	
C17	0.8521 (3)	0.1185 (5)	0.7795 (2)	0.0761 (11)	
H17	0.8701	0.0584	0.7469	0.091*	
C18	0.8431 (2)	0.0720 (3)	0.8490 (2)	0.0590 (9)	
H18	0.8551	−0.0194	0.8625	0.071*	
C19	0.6045 (2)	0.1174 (3)	0.9649 (2)	0.0571 (9)	
C20	0.4795 (2)	0.0390 (3)	0.8630 (2)	0.0541 (9)	
C21	0.3936 (2)	0.0089 (4)	0.8024 (2)	0.0681 (11)	
H21A	0.4025	0.0140	0.7514	0.082*	
H21B	0.3736	−0.0830	0.8095	0.082*	
C22	0.2436 (2)	0.0887 (3)	0.7565 (2)	0.0503 (8)	
C23	0.1754 (2)	0.1615 (3)	0.7739 (2)	0.0541 (9)	
H23	0.1882	0.2155	0.8183	0.065*	
C24	0.0885 (2)	0.1538 (3)	0.7253 (2)	0.0561 (9)	
H24	0.0428	0.2034	0.7366	0.067*	
C25	0.0695 (2)	0.0727 (3)	0.6602 (2)	0.0526 (9)	
C26	0.1366 (2)	−0.0011 (4)	0.6430 (2)	0.0639 (10)	
H26	0.1232	−0.0560	0.5989	0.077*	
C27	0.2241 (2)	0.0064 (4)	0.6917 (2)	0.0615 (10)	
H27	0.2696	−0.0440	0.6805	0.074*	

### Atomic displacement parameters (Å^2^)

**Table:**

	*U*^11^	*U*^22^	*U*^33^	*U*^12^	*U*^13^	*U*^23^
Sn1	0.04381 (17)	0.04862 (16)	0.04831 (17)	−0.00257 (11)	0.00596 (11)	−0.00390 (10)
Br1	0.0507 (3)	0.1201 (4)	0.0662 (3)	0.0068 (2)	−0.0046 (2)	0.0018 (2)
S1	0.0507 (6)	0.0825 (6)	0.0681 (6)	0.0058 (5)	0.0096 (5)	−0.0185 (5)
S2	0.0428 (5)	0.0659 (6)	0.0766 (7)	0.0112 (5)	0.0106 (5)	−0.0134 (5)
O1	0.0415 (14)	0.0626 (14)	0.0735 (17)	0.0067 (12)	0.0047 (12)	−0.0141 (13)
N1	0.0398 (16)	0.0599 (17)	0.0642 (19)	0.0036 (15)	0.0082 (15)	−0.0037 (15)
N2	0.0459 (18)	0.0541 (17)	0.075 (2)	0.0067 (15)	0.0116 (16)	−0.0043 (16)
C1	0.0456 (19)	0.0422 (17)	0.051 (2)	−0.0010 (16)	0.0094 (16)	−0.0037 (15)
C2	0.054 (2)	0.067 (2)	0.052 (2)	−0.0032 (19)	0.0142 (18)	−0.0060 (18)
C3	0.052 (2)	0.075 (3)	0.083 (3)	−0.016 (2)	0.018 (2)	−0.016 (2)
C4	0.064 (3)	0.069 (2)	0.073 (3)	−0.013 (2)	−0.001 (2)	−0.018 (2)
C5	0.104 (3)	0.072 (3)	0.044 (2)	−0.014 (3)	0.002 (2)	−0.006 (2)
C6	0.080 (3)	0.070 (2)	0.051 (2)	−0.024 (2)	0.016 (2)	−0.0039 (19)
C7	0.044 (2)	0.0523 (18)	0.0446 (19)	0.0023 (16)	0.0125 (16)	−0.0013 (15)
C8	0.049 (2)	0.052 (2)	0.073 (2)	−0.0048 (19)	−0.0012 (18)	−0.0059 (19)
C9	0.055 (2)	0.050 (2)	0.101 (3)	−0.002 (2)	−0.001 (2)	−0.005 (2)
C10	0.068 (3)	0.050 (2)	0.079 (3)	0.008 (2)	0.016 (2)	0.004 (2)
C11	0.043 (2)	0.069 (2)	0.063 (2)	0.017 (2)	0.0123 (18)	0.0032 (19)
C12	0.0399 (19)	0.061 (2)	0.060 (2)	0.0001 (18)	0.0101 (17)	−0.0073 (18)
C13	0.0360 (18)	0.0492 (18)	0.051 (2)	−0.0021 (15)	0.0031 (15)	−0.0020 (16)
C14	0.070 (3)	0.055 (2)	0.070 (3)	0.005 (2)	0.004 (2)	−0.001 (2)
C15	0.077 (3)	0.068 (3)	0.081 (3)	−0.011 (2)	−0.007 (2)	0.024 (2)
C16	0.069 (3)	0.112 (4)	0.055 (2)	−0.024 (3)	0.007 (2)	0.012 (3)
C17	0.071 (3)	0.098 (3)	0.063 (3)	−0.010 (3)	0.025 (2)	−0.009 (2)
C18	0.058 (2)	0.057 (2)	0.060 (2)	−0.0044 (18)	0.0140 (19)	0.0003 (18)
C19	0.048 (2)	0.058 (2)	0.060 (2)	0.0054 (18)	0.0094 (18)	−0.0035 (18)
C20	0.044 (2)	0.0504 (18)	0.064 (2)	0.0034 (17)	0.0102 (18)	−0.0033 (17)
C21	0.050 (2)	0.063 (2)	0.085 (3)	0.0012 (19)	0.011 (2)	−0.017 (2)
C22	0.0373 (18)	0.0498 (19)	0.059 (2)	−0.0015 (16)	0.0064 (16)	−0.0007 (17)
C23	0.048 (2)	0.0521 (19)	0.061 (2)	−0.0003 (17)	0.0152 (18)	−0.0091 (17)
C24	0.046 (2)	0.059 (2)	0.062 (2)	0.0056 (18)	0.0134 (18)	0.0024 (18)
C25	0.043 (2)	0.057 (2)	0.053 (2)	0.0025 (17)	0.0071 (17)	0.0107 (17)
C26	0.059 (2)	0.069 (2)	0.055 (2)	−0.002 (2)	0.0044 (19)	−0.0086 (19)
C27	0.046 (2)	0.069 (2)	0.065 (2)	0.0097 (19)	0.0092 (19)	−0.014 (2)

### Geometric parameters (Å, °)

**Table:**

Sn1—C13	2.130 (3)	C9—H9	0.9300
Sn1—C7	2.146 (3)	C10—C11	1.377 (5)
Sn1—C1	2.149 (3)	C10—H10	0.9300
Sn1—S1	2.4721 (10)	C11—C12	1.388 (5)
Sn1—N1	2.919 (3)	C11—H11	0.9300
Br1—C25	1.892 (4)	C12—H12	0.9300
S1—C19	1.738 (4)	C13—C18	1.376 (4)
S2—C19	1.724 (4)	C13—C14	1.384 (4)
S2—C20	1.725 (4)	C14—C15	1.393 (5)
O1—C22	1.370 (4)	C14—H14	0.9300
O1—C21	1.404 (4)	C15—C16	1.358 (6)
N1—C19	1.299 (4)	C15—H15	0.9300
N1—N2	1.375 (4)	C16—C17	1.357 (6)
N2—C20	1.301 (4)	C16—H16	0.9300
C1—C2	1.375 (4)	C17—C18	1.376 (5)
C1—C6	1.378 (4)	C17—H17	0.9300
C2—C3	1.384 (5)	C18—H18	0.9300
C2—H2	0.9300	C20—C21	1.485 (5)
C3—C4	1.360 (5)	C21—H21A	0.9700
C3—H3	0.9300	C21—H21B	0.9700
C4—C5	1.373 (5)	C22—C27	1.376 (5)
C4—H4	0.9300	C22—C23	1.386 (4)
C5—C6	1.389 (5)	C23—C24	1.376 (4)
C5—H5	0.9300	C23—H23	0.9300
C6—H6	0.9300	C24—C25	1.375 (5)
C7—C12	1.382 (4)	C24—H24	0.9300
C7—C8	1.385 (5)	C25—C26	1.374 (5)
C8—C9	1.385 (5)	C26—C27	1.386 (5)
C8—H8	0.9300	C26—H26	0.9300
C9—C10	1.360 (5)	C27—H27	0.9300
			
C13—Sn1—C7	115.65 (11)	C18—C13—Sn1	120.5 (2)
C13—Sn1—C1	108.20 (12)	C14—C13—Sn1	121.9 (3)
C7—Sn1—C1	106.47 (12)	C13—C14—C15	120.6 (4)
C13—Sn1—S1	109.32 (9)	C13—C14—H14	119.7
C7—Sn1—S1	116.71 (8)	C15—C14—H14	119.7
C1—Sn1—S1	98.76 (9)	C16—C15—C14	120.3 (4)
C19—S1—Sn1	93.52 (12)	C16—C15—H15	119.8
C19—S2—C20	86.94 (17)	C14—C15—H15	119.8
C22—O1—C21	116.7 (3)	C17—C16—C15	119.6 (4)
C19—N1—N2	112.9 (3)	C17—C16—H16	120.2
C20—N2—N1	112.2 (3)	C15—C16—H16	120.2
C2—C1—C6	117.8 (3)	C16—C17—C18	120.7 (4)
C2—C1—Sn1	120.2 (2)	C16—C17—H17	119.7
C6—C1—Sn1	121.9 (2)	C18—C17—H17	119.7
C1—C2—C3	121.6 (3)	C17—C18—C13	121.3 (4)
C1—C2—H2	119.2	C17—C18—H18	119.3
C3—C2—H2	119.2	C13—C18—H18	119.3
C4—C3—C2	119.8 (4)	N1—C19—S2	113.8 (3)
C4—C3—H3	120.1	N1—C19—S1	121.7 (3)
C2—C3—H3	120.1	S2—C19—S1	124.4 (2)
C3—C4—C5	120.1 (4)	N2—C20—C21	121.3 (3)
C3—C4—H4	120.0	N2—C20—S2	114.1 (3)
C5—C4—H4	120.0	C21—C20—S2	124.5 (3)
C4—C5—C6	119.7 (3)	O1—C21—C20	109.4 (3)
C4—C5—H5	120.2	O1—C21—H21A	109.8
C6—C5—H5	120.2	C20—C21—H21A	109.8
C1—C6—C5	121.1 (3)	O1—C21—H21B	109.8
C1—C6—H6	119.5	C20—C21—H21B	109.8
C5—C6—H6	119.5	H21A—C21—H21B	108.2
C12—C7—C8	118.4 (3)	O1—C22—C27	124.3 (3)
C12—C7—Sn1	116.5 (2)	O1—C22—C23	115.7 (3)
C8—C7—Sn1	125.0 (3)	C27—C22—C23	120.0 (3)
C7—C8—C9	119.9 (4)	C24—C23—C22	120.0 (3)
C7—C8—H8	120.1	C24—C23—H23	120.0
C9—C8—H8	120.1	C22—C23—H23	120.0
C10—C9—C8	121.4 (4)	C25—C24—C23	119.9 (3)
C10—C9—H9	119.3	C25—C24—H24	120.0
C8—C9—H9	119.3	C23—C24—H24	120.0
C9—C10—C11	119.5 (3)	C26—C25—C24	120.5 (3)
C9—C10—H10	120.3	C26—C25—Br1	119.4 (3)
C11—C10—H10	120.3	C24—C25—Br1	120.1 (3)
C10—C11—C12	119.6 (3)	C25—C26—C27	119.8 (3)
C10—C11—H11	120.2	C25—C26—H26	120.1
C12—C11—H11	120.2	C27—C26—H26	120.1
C7—C12—C11	121.2 (3)	C22—C27—C26	119.8 (3)
C7—C12—H12	119.4	C22—C27—H27	120.1
C11—C12—H12	119.4	C26—C27—H27	120.1
C18—C13—C14	117.5 (3)		
			
C13—Sn1—S1—C19	63.59 (15)	C1—Sn1—C13—C14	−66.9 (3)
C7—Sn1—S1—C19	−70.03 (16)	S1—Sn1—C13—C14	39.7 (3)
C1—Sn1—S1—C19	176.47 (15)	C18—C13—C14—C15	−0.6 (5)
C19—N1—N2—C20	0.0 (4)	Sn1—C13—C14—C15	175.4 (3)
C13—Sn1—C1—C2	−8.8 (3)	C13—C14—C15—C16	−0.1 (6)
C7—Sn1—C1—C2	116.1 (3)	C14—C15—C16—C17	0.7 (7)
S1—Sn1—C1—C2	−122.5 (3)	C15—C16—C17—C18	−0.7 (7)
C13—Sn1—C1—C6	174.5 (3)	C16—C17—C18—C13	0.0 (6)
C7—Sn1—C1—C6	−60.6 (3)	C14—C13—C18—C17	0.6 (5)
S1—Sn1—C1—C6	60.8 (3)	Sn1—C13—C18—C17	−175.4 (3)
C6—C1—C2—C3	0.6 (5)	N2—N1—C19—S2	0.1 (4)
Sn1—C1—C2—C3	−176.3 (3)	N2—N1—C19—S1	−178.5 (2)
C1—C2—C3—C4	0.4 (6)	C20—S2—C19—N1	−0.1 (3)
C2—C3—C4—C5	−0.7 (6)	C20—S2—C19—S1	178.4 (3)
C3—C4—C5—C6	−0.1 (6)	Sn1—S1—C19—N1	7.6 (3)
C2—C1—C6—C5	−1.3 (6)	Sn1—S1—C19—S2	−170.8 (2)
Sn1—C1—C6—C5	175.4 (3)	N1—N2—C20—C21	177.7 (3)
C4—C5—C6—C1	1.1 (6)	N1—N2—C20—S2	−0.1 (4)
C13—Sn1—C7—C12	104.7 (2)	C19—S2—C20—N2	0.1 (3)
C1—Sn1—C7—C12	−15.5 (3)	C19—S2—C20—C21	−177.6 (3)
S1—Sn1—C7—C12	−124.6 (2)	C22—O1—C21—C20	−178.6 (3)
C13—Sn1—C7—C8	−75.0 (3)	N2—C20—C21—O1	−169.3 (3)
C1—Sn1—C7—C8	164.8 (3)	S2—C20—C21—O1	8.3 (5)
S1—Sn1—C7—C8	55.7 (3)	C21—O1—C22—C27	−14.0 (5)
C12—C7—C8—C9	0.6 (5)	C21—O1—C22—C23	165.3 (3)
Sn1—C7—C8—C9	−179.7 (3)	O1—C22—C23—C24	179.2 (3)
C7—C8—C9—C10	−0.2 (6)	C27—C22—C23—C24	−1.5 (5)
C8—C9—C10—C11	−0.9 (6)	C22—C23—C24—C25	0.8 (5)
C9—C10—C11—C12	1.6 (6)	C23—C24—C25—C26	0.1 (5)
C8—C7—C12—C11	0.1 (5)	C23—C24—C25—Br1	179.8 (2)
Sn1—C7—C12—C11	−179.6 (2)	C24—C25—C26—C27	−0.1 (5)
C10—C11—C12—C7	−1.2 (5)	Br1—C25—C26—C27	−179.9 (3)
C7—Sn1—C13—C18	−10.3 (3)	O1—C22—C27—C26	−179.3 (3)
C1—Sn1—C13—C18	109.0 (3)	C23—C22—C27—C26	1.4 (5)
S1—Sn1—C13—C18	−144.5 (3)	C25—C26—C27—C22	−0.6 (6)
C7—Sn1—C13—C14	173.8 (3)		

### Hydrogen-bond geometry (Å, °)

**Table:**

*D*—H···*A*	*D*—H	H···*A*	*D*···*A*	*D*—H···*A*
C9—H9···Br1^i^	0.93	2.87	3.627 (4)	139
C8—H8···N1	0.93	2.54	3.274 (5)	136

Symmetry codes: (i) −*x*+1/2, *y*−1/2, −*z*+3/2.
